# Complete genome sequence of the lytic vB_EcoP_ShWW44 bacteriophage, effective against bovine pathogenic *Escherichia coli*

**DOI:** 10.1128/mra.00195-25

**Published:** 2025-06-12

**Authors:** Pavel G. Alexyuk, Andrey P. Bogoyavlenskiy, Kuralay S. Akanova, Timur T. Kerimov, Adolat N. Manakbayeva, Yergali S. Moldakhanov, Igor D. Yershov, Madina S. Alexyuk

**Affiliations:** 1 Research and Production Center for Microbiology and Virology 374530 , Almaty, Kazakhstan; Portland State University, Portland, Oregon, USA

**Keywords:** bacteriophage, sequencing, genome analysis, bacteriophage therapy

## Abstract

Here, we report the genome sequence of *Escherichia* lytic phage vB_EcoP_ShWW44, a podovirus isolated from wastewater using bovine *Escherichia coli* as a host. The genome is 72,444 bp long and has 89 predicted coding sequences, including an alanine-tRNA gene. It belongs to the subfamily *Enquatrovirinae*, genus *Gamaleyavirus*.

## ANNOUNCEMENT

Infections with antibiotic-resistant *Escherichia coli* are one of the major problems of modern animal husbandry. To solve this problem, alternative control methods to antibiotic therapy are needed, which has revived researchers’ interest in bacteriophages and phage therapy (1–4). The current study reports the complete genome of the vB_EcoP_ShWW44 phage, capable of lysing pathogenic for calves, antibiotic-resistant *E. coli*.

vB_EcoP_ShWW44 was isolated by enriching 10 mL of filtered (0.45 µm syringe filter) Shymkent city wastewater (42°24'30.9"N 69°30'05.9"E) collected in 2023 with 1 mL (10^8^ CFU/mL) of bovine *E. coli* isolate (strain 44), adding 1 mL of 10× Trypticasein soy broth (Condalab, Spain) and incubating the sample for 18 h at 37°C. After incubation, the enriched sample was centrifuged at 4,500 × *g* for 15 min and filtered through 0.45 µm PES syringe filters (LLG-Labware, Germany). The filtrate was tested for phage activity using the double overlay agar method (5). Bacteriophage was purified from a clear single plaque isolate using the soft agar overlay method (6). The phage was propagated by co-cultivation with *E. coli* strain 44 (10^8^ CFU/mL, MOI ratio of 1) at 37°C for 18 h. Before the concentration of phage particles using VivaSpin centrifugal concentrators (Sartorius, Germany, 30 kDa PES membrane) (7), bacterial cells were removed by centrifugation and filtration as described above. To remove residual bacterial DNA and RNA, the obtained concentrate was treated with RNase A (10 mg/mL) and DNase I (1 U/µL) (8). The PureLink viral DNA/RNA minikit (Thermo Fisher Scientific) was used to extract phage genomic DNA according to the manufacturer’s instructions. One nanogram of DNA was used for library preparation with the Nextera XT DNA Library Preparation Kit (Illumina). Whole-genome sequencing was performed on the Illumina MiSeq instrument using the MiSeq v3 2 × 300 bp (600 cycles) reagent kit (Illumina) with an average depth of coverage of 231×. A total of 1,010,240 paired-end (PE) reads were generated. The raw reads were quality controlled using FastQC v0.12.1 (9) and trimmed with Trimmomatic 0.38.0 (10). *De novo* assembly was performed from 910,334 PE reads with SPAdes version 3.12.0 (11), which resulted in a contig of 72,444 bp. Phage genome termini and genome completeness were determined using PhageTerm (Galaxy Version 1.0.12) (12) and CheckV version 0.8.1 from PhageScope (13). Predicted coding sequences were annotated using PROKKA 1.7 (14) and manually edited. The coding regions of tRNAs were identified using tRNAscan-SE (15). The lytic lifestyle of the phage was predicted using BACPHLIP 0.9.6 (16). The genomic similarity of vB_EcoP_ShWW44 to other members of the genus was analyzed using the VICTOR algorithm (17). All tools were run with default parameters.

**Fig 1:**
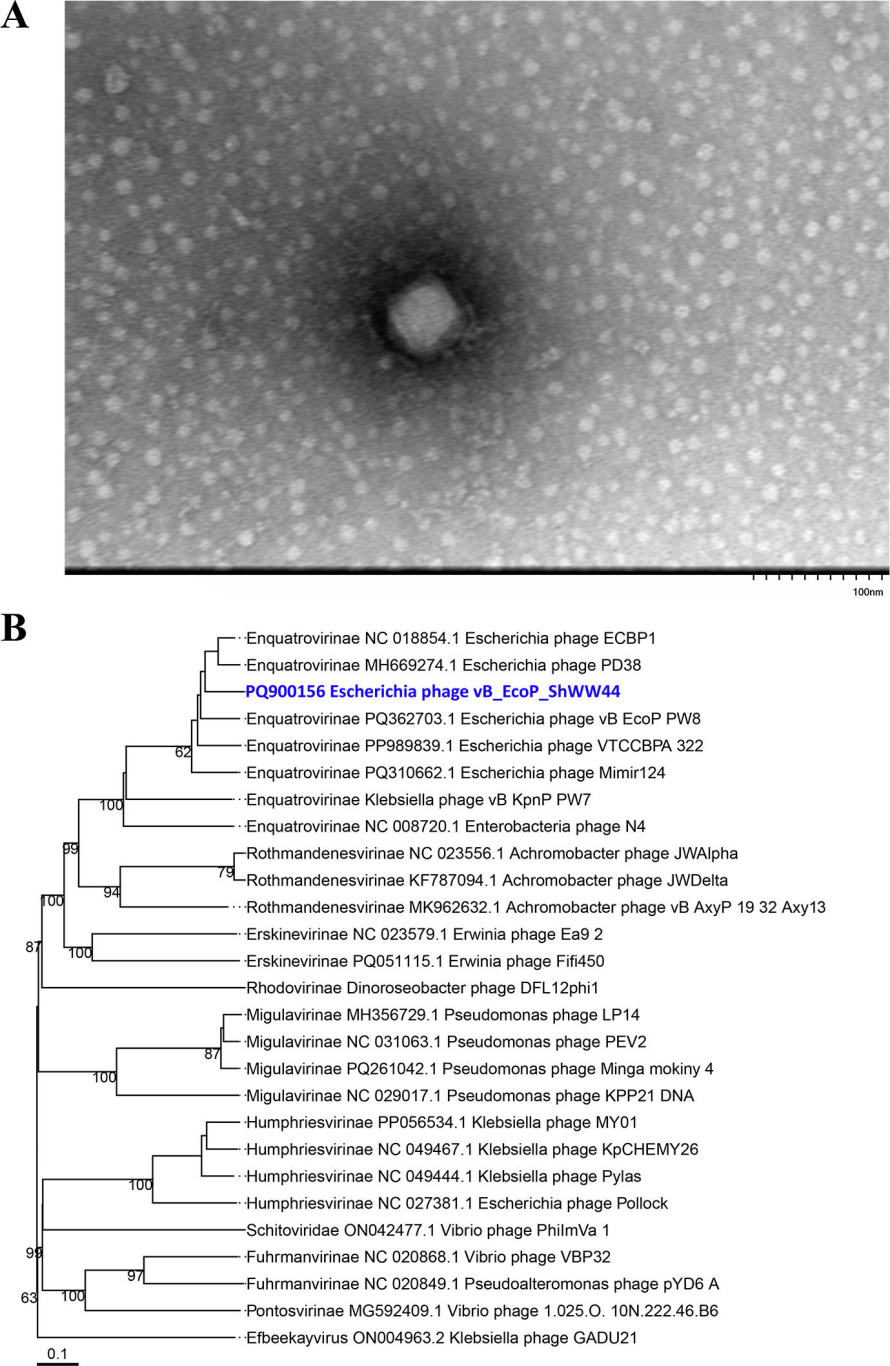
(**A**) Transmission electron micrograph of vB_EcoP_ShWW44 (Phage sample was prepared for imaging as in reference 18. In brief, 3% phosphotungstic acid [pH 6.8] was used to stain the sample, and the grids were observed at an instrumental magnification of 60–120 thousand times and a voltage of 80 kV using HT7800 “Hitachi” microscope [Japan]). (**B**) Phylogenetic tree generated by the VICTOR web service (https://victor.dsmz.de; accessed on 27 January 2025), a method for the genome-based phylogeny and classification of prokaryotic viruses. All pairwise comparisons of the nucleotide sequences were conducted using the Genome-BLAST Distance Phylogeny method under settings recommended for prokaryotic viruses (19).

Transmission electron microscopy revealed that vB_EcoP_ShWW44 has an icosahedral capsid with a diameter of about 50 nm and a short tail (Fig. 1A). vB_EcoP_ShWW44 possesses a linear double-stranded DNA genome of 72,444 bp with a GC content of 42.7%. Genome annotation predicts 89 coding features, of which 37 have assigned functions, including tRNA(Ala) (Table 1). BACPHLIP analysis predicted the phage to be lytic. Phylogenetic analysis established that the investigated phage belongs to the subfamily *Enquatrovirinae*, genus *Gamaleyavirus* (Fig. 1B).

**TABLE 1:** Genome details and comparative analysis of the vB_EcoP_ShWW44 bacteriophage with related phages

Feature		vB_EcoP_ShWW44	
Genome size, bp		72,444	
CheckV completeness (%)		100	
CheckV quality		High quality	
ORF number^*a*^		89	
GC content		42.7%	
tRNAs		1	
Morphology		Podovirus	
Bacphlip life cycle (%)		Virulent (99.4)	

Closest relatives	*Escherichia phage St11Ph5*	*Escherichia phage PD38*	*Escherichia phage ECBP1*
GenBank accession no.	NC_070873.1	MH669274.1	NC_018854.1
Percent identity (%)	94.72	94.78	94.98
Query coverage (%)	100	85	86
Subfamily	*Enquatrovirinae*	*Enquatrovirinae*	*Enquatrovirinae*
*Genus*	*Gamaleyavirus*	*Gamaleyavirus*	*Gamaleyavirus*

^*a*^ ORF, Open Reading Frame. .

## Data Availability

The vB_EcoP_ShWW44 genome is available in GenBank with accession number PQ900156. The raw sequence reads are available at NCBI SRA database with accession number SRR32240720.
